# Protective effects of 5-aminolevulinic acid on heat stress in bovine mammary epithelial cells

**DOI:** 10.5713/ajas.20.0349

**Published:** 2020-08-24

**Authors:** Md Aminul Islam, Yoko Noguchi, Shin Taniguchi, Shinichi Yonekura

**Affiliations:** 1 Graduate School of Medicine, Science and Technology, Shinshu University, Kamiina, Nagano 399-4598, Japan; 2 Neopharma Japan Co., Ltd. Tokyo 102-0071, Japan; 3 Graduate School of Biosphere Science, Hiroshima University, Higashi-Hiroshima 739-8528, Japan; 4 Department of Biomolecular Innovation, Institute for Biomedical Sciences, Interdisciplinary Cluster for Cutting Edge Research, Shinshu University, Kamiina, Nagano 399-4598, Japan

**Keywords:** Heat Stress, 5-Aminolevulinic Acid, Bovine Mammary Epithelial Cells, Endoplasmic Reticulum Stress, Oxidative Stress

## Abstract

**Objective:**

Cells have increased susceptibility to activation of apoptosis when suffering heat stress (HS). An effective supplementation strategy to mimic heat-induced apoptosis of bovine mammary epithelial cells (MECs) is necessary to maintain optimal milk production. This study aimed to investigate possible protective effects of the anti-apoptotic activity of 5-aminolevulinic acid (5-ALA) against HS-induced damage of bovine MECs.

**Methods:**

Bovine MECs were pretreated with or without 5-ALA at concentrations of 10, 100, and 500 μM for 24 h followed by HS (42.5°C for 24 h and 48 h). Cell viability was measured with 3-(4,5-dimethylthiazol-2-yl)-2,5-diphenyltetrazolium bromide assays. Real-time quantitative polymerase chain reaction and Western blotting were used to explore the regulation of genes associated with apoptosis, oxidative stress, and endoplasmic reticulum (ER) stress genes.

**Results:**

We found that 5-ALA induces cytoprotection via inhibition of apoptosis markers after HS-induced damage. Pretreatment of bovine MECs with 5-ALA resulted in dramatic upregulation of mRNA for nuclear factor erythroid-derived 2-like factor 2, heme oxygenase-1, and NAD(P)H quinone oxidoreductase 1, all of which are antioxidant stress genes. Moreover, 5-ALA pretreatment significantly suppressed HS-induced ER stress-associated markers, glucose-regulated protein 78, and C/EBP homologous protein expression levels.

**Conclusion:**

5-ALA can ameliorate the ER stress in heat stressed bovine MEC via enhancing the expression of antioxidant gene.

## INTRODUCTION

Global warming is becoming more severe and can affect the productivity of dairy cattle. Heat stress (HS) produces negative effects on the physiology of lactation, growth, and reproduction of livestock species compared with neutral thermal conditions. HS increases body temperature and reduces feed intake and milk production, resulting in economic losses for the global dairy industry [1,2]. Experiments involving controlled dry matter intake under HS conditions have revealed that reduced nutrient intake accounts for only 50% of HS-induced decrease in milk yield [3]. Thus, factors other than reduced feed intake caused by HS contribute to decreased milk yield.

The bovine mammary gland is composed of secretory tissue embedded in a layer of mammary epithelial cells (MECs). These cells are the main functional units for milk production. Numbers of MECs and their secretory activity determine the amount of milk produced during lactation [4]. The most important cause of decreased milk yield is an alteration of the secretory function of mammary glands caused by HS [5]. Cells have increased susceptibility to apoptosis when suffering HS [6]. HS is also an environmental factor responsible for inducing oxidative stress and subsequent cellular damage. Heat induced excessive reactive oxygen species (ROS) (marker of oxidative stress) production in bovine MECs provides direct evidence of a redox imbalance status which impairs the cellular antioxidant capacity [7]. Disruption of cellular antioxidant capacity as well as overproduction of ROS can upset endoplasmic reticulum (ER) homeostasis and impair protein folding leading to induce ER stress [8]. Due to imbalanced homeostasis an adaptive network of signaling cascades known as unfolded protein response (UPR) is activated [8]. UPR consists of three arms: PKR-like endoplasmic reticulum kinase (PERK), inositol-requiring enzyme 1 (IRE1), and activating transcription factor 6 (ATF6) [9]. The dominant PERK arm over ATF6 and IRE1/XBP-1 signaling pathways is essential for upregulation of proapoptotic transcriptional factor C/EBP homologous protein (CHOP) under ER stress conditions [10]. Accumulating evidence indicates that ER stress-mediated apoptotic cell death plays a critical role in HS-induced cellular damage [7,11]. Our previous study proved that ER stress-induced CHOP is negatively correlated with milk yield in mammary gland tissue [12]. Thus, an effective supplementation strategy to counter heat load-induced apoptosis in MECs is necessary for maintaining optimal milk production.

5-Aminolevulinic acid (5-ALA) is a natural non-alpha amino acid found in foods, such as spinach, green peppers, tomatoes, shiitake mushrooms, bananas, and potatoes. Larger amounts are found in fermented products, such as wine, vinegar, sake, and soy sauce. 5-ALA is a precursor for biosynthetic tetrapyrroles, such as chlorophyll, vitamin B_12_, and heme [13]. Glycine, a non-essential amino acid, combines with succinyl-CoA to form 5-ALA in the presence of 5-ALA synthase as part of the heme biosynthetic pathway in animals [14]. 5-ALA can reduce nephrotoxicity and apoptosis in murine tubular epithelial cells and can protect kidneys from acute injury [15,16]. Moreover, both *in vivo* and *in vitro* studies show that 5-ALA can induce heme oxygenase-1 (HO-1) expression in kidney cells [17,18]. Enhanced expression of HO-1 protects against oxidative and other stresses, such as cisplatin-induced nephrotoxicity, hydrogen peroxide-induced cardiomyocyte hypertrophy, and ischemia-reperfusion-induced renal injury [16,18–20]. Therefore, this study aimed to investigate the possible protective effects of 5-ALA against HS-induced damage to bovine MECs. In this current research, we assessed the MAC-T cell (a MEC line) viability after pretreatment with 5-ALA followed by HS. We also evaluate ER and oxidative stress marker gene expression in heat stressed MAC-T cell with and without 5-ALA treatment. Furthermore, we investigate the expression of ER stress marker protein.

## MATERIALS AND METHODS

### Chemicals and reagents

5-ALA was provided by Neopharma Japan Co. Ltd. (Tokyo, Japan). Dulbecco’s modified eagle medium (DMEM) was purchased from Invitrogen (Carlsbad, CA, USA). Fetal bovine serum (FBS) was obtained from Equitech-Bio (Cotton Gin Lane, Kerrville, TX, USA). Penicillin, streptomycin, hydrocortisone, and bovine insulin were purchased from Sigma-Aldrich (St. Louis, MO, USA). All other compounds were obtained from Nacalai Tesque (Kyoto, Japan).

### Cell culture and heat stress treatment

Immortalized bovine MECs (MAC-T cells) were generously provided by Dr. Sangun Roh of Tohoku University, Sendai, Japan. Bovine MECs of passage 35 were cultured in DMEM containing 10% FBS, 1% penicillin and streptomycin, 5 μg/mL bovine insulin, and 1 μg/mL hydrocortisone. Cells were maintained in a humidified incubator at 37°C in an atmosphere of 5% CO_2_. We applied modified methods of heat treatment; 42.5°C temperature for 24 h or 48 h for HS in MAC-T cell which was adopted from previous study [7,21]. Cells cultured at 37°C were used as controls. 5-ALA was added to bovine MECs for 24 h before heat treatment to examine its activity in preventing HS-related damage.

### Cell viability and 5-ALA toxicity determination

Cell viability and 5-ALA toxicity evaluations were performed using an MTT Cell Viability Assay Kit (Biotium, Fremont, CA, USA) following the manufacturer’s protocol. Briefly, bovine MECs were seeded in 96-well plates at a density of 1×10^4^ cells/well and were cultured for 24 h to reach an optimal density. Various concentrations of 5-ALA (0, 50, 100, 250, and 500 μM) were added to cells, and incubation continued for 48 h to test for 5-ALA toxicity. Different doses of 5-ALA were determined based on the knowledge of previous study [18]. Bovine MECs were pretreated with 5-ALA (10, 100, and 500 μM) followed by HS challenged for 48 h to examine 5-ALA effects on cellular damage caused by HS. After incubation, 10 μL of the 3-(4,5-dimethylthiazol-2-yl)- 2,5-diphenyltetrazolium bromide (MTT) solution was added to 100 μL of culture medium. After 4 h of incubation at 37°C, 200 μL of dimethylsulfoxide was added to each well. Absorbance was measured at 570 nm and a reference wavelength of 630 nm using a multimode microplate reader (iMark microplate reader, Bio-Rad, Hercules, CA, USA).

### RNA extraction and quantitative real-time polymerase chain reaction

Total RNA was isolated from bovine MECs using TRIzol (Invitrogen, USA), according to the manufacturer’s protocol. For quantitative real-time (qRT) polymerase chain reaction (PCR), cDNA was synthesized from total RNA using a qPCR RT Master Mix with gDNA Remover (Toyobo, Osaka, Japan). The qRT-PCR used a SYBR Premix Ex Taq II (TaKaRa Biotechnology, Kusatsu, Japan). Primers used for quantitative PCR are shown in Table 1. Relative gene expression was quantified using the 2^−ΔΔCT^ comparative method and is represented as values relative to control. β-Actin (*ACTB*) was used as the reference gene. The sensitivity of reactions and amplification of contaminating products, such as extensions of self-annealed primers, were evaluated by amplifying serial cDNA dilutions. The specificity of amplified PCR products is analysed by the dissociation curve and agarose gel electrophoresis. Data analyses followed the manufacturer’s instructions.

### Western blot analysis

Cells were washed twice and then lysed with radioimmunoprecipitation assay lysis buffer (50 m*M* Tris-HCl, 150 m*M* NaCl pH 7.4, 0.05% sodium dodecyl sulfate (SDS), 0.2% sodium deoxycholate, 1 mM ethylenediaminetetraacetic acid, 1% Tergitol-type NP-40) containing protease inhibitor cocktail (Nacalai Tesque, Japan). Following centrifugation (10 min at 20,000×g), protein concentrations were determined using a Bio-Rad Protein Assay Kit (Bio-Rad Laboratories, Hercules, CA, USA). Cell extracts (60 μg) were subjected to SDS-polyacrylamide gel electrophoresis on 4% to 20% polyacrylamide gels and were transferred to polyvinylidene difluoride membranes. Membranes were incubated for 1 h in 1% PBT and 0.01% Tween 20 solution containing 4% skim milk powder (blocking buffer). Our previous study established that ER stress induced apoptosis enhance the p-PERK, CHOP, and cleaved caspase-3 protein expression in bovine MECs [22]. Therefore, we measured PERK, p-PERK, CHOP, and cleaved caspase-3 protein expression level using western blot analysis. Thus, membranes were then incubated with anti-phosphorylated PERK (Santa Cruz Biotechnology, Santa Cruz, CA, USA), anti-PERK (Santa Cruz), anti-CHOP (Life Span Bioscience, Inc., Seattle, WA, USA), anti-cleaved caspase-3 (Cell Signaling, Danvers, MA, USA), and anti-α-tubulin (MBL Co., Nagoya, Japan) antibodies diluted in blocking buffer at room temperature. Next, the membranes were incubated with anti-rabbit immunoglobulin G secondary antibody (GE Healthcare, Pittsburgh, PA, USA), and enhanced chemiluminescent (ECL) membranes were visualized using an ECL Prime Western Blotting Detection Reagent Kit (GE Healthcare, USA) and images obtained using an Image Quant LAS 500 (GE Healthcare, USA).

### Statistical analysis

Values are expressed as the mean±standard error of the mean with at least three replicates in each experimental group. Tukey-Kramer test or Student’s *t*-test were used to assess statistical differences between groups. Differences were considered significant at p<0.05.

## RESULTS

### 5-ALA prevents HS reduced cell viability in bovine MECs

**Figure 1:**
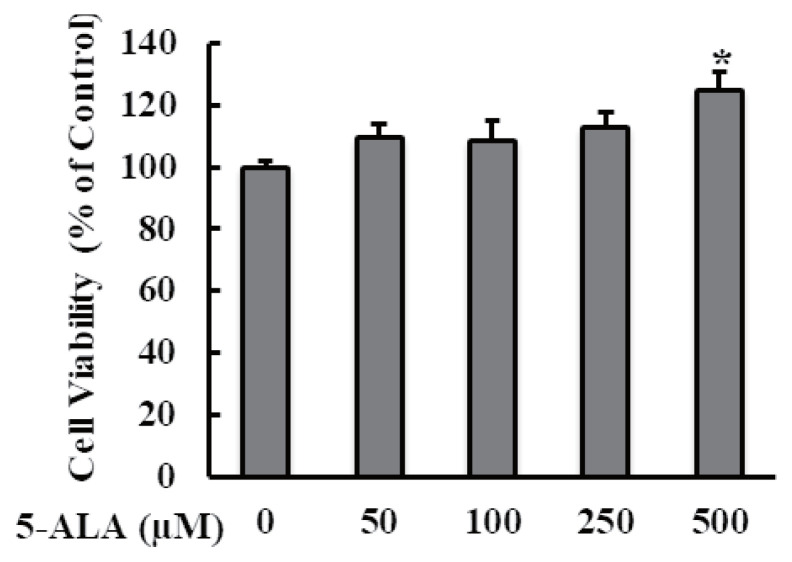
Effect of different doses of 5-ALA on bovine MEC viability. Confluent bovine MECs were treated with 50, 100, 250, and 500 μM of 5-ALA for 48 h. Cell viability was measured using MTT assays. Absorbance was measured at 570 and 630 nm using a multimode microplate reader to calculate survival rates expressed as the percentage of the control cells without 5-ALA treatment. Data are presented as mean± standard error of the mean for three independent experiments. 5-ALA, 5-aminolevulinic acid; MEC, mammary epithelial cells; MTT, 3-(4,5-dimethylthiazol-2-yl)-2,5-diphenyltetrazolium bromide. * Indicates p<0.05 compared with the control.

**Figure 2:**
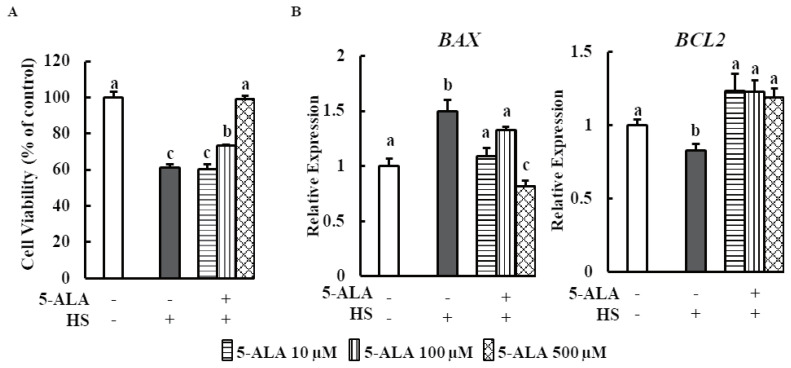
5-ALA counters HS reduced cell viability in bovine MECs. (A) Bovine MECs were pretreated with or without 5-ALA at concentrations of 10, 100, and 500 μM for 24 h followed by HS (42.5°C for 48 h). Cell viability was measured by MTT assay and expressed the percentage of control cells continuously cultured at 37°C and received no 5-ALA treatment. (B) Bovine MECs were pretreated with or without 5-ALA at concentrations of 10, 100, and 500 μM for 24 h followed by HS (42.5°C for 48 h). mRNA levels of *BAX* and *BCL2* were determined by RT-qPCR and normalized to *ACTB* levels. Data are presented as mean±standard error of the mean for three independent experiments. 5-ALA, 5-aminolevulinic acid; HS, heat stress; MEC, mammary epithelial cells; RT-qPCR, real-time quantitative polymerase chain reaction; MTT, 3-(4,5-dimethylthiazol-2-yl)-2,5-diphenyltetrazolium bromide. ^a–c^ Means with different letters are significantly different, p<0.05.

Cytotoxicity of 5-ALA was evaluated following 48 h treatment of bovine MECs with 5-ALA concentrations of 50, 100, 250, and 500 μM, and no cytotoxic effect was found (Figure 1). HS significantly reduced bovine MEC viability (Figure 2A).

We investigated the effects of 5-ALA treatment on bovine MEC viability after HS. Cell viability markedly increased when cells were pretreated with 5-ALA at concentrations of 100 and 500 μM. Real-time qPCR analysis showed that expression of anti-apoptosis gene B-cell lymphoma 2 (*BCL2*) was downregulated and expression of pro-apoptosis gene BCL2-associated X (*BAX*) was upregulated in heat-stressed bovine MECs. Pretreatment with 5-ALA (10, 100, and 500 μM) resulted in a significant reduction of *BAX* expression and an increase in *BCL2* expression (Figure 2B).

### Effect of 5-ALA on the expression of oxidative stress-related gene during HS

**Figure 3:**
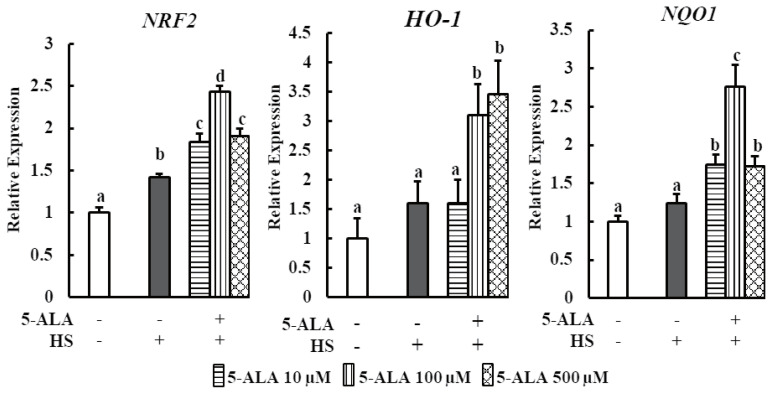
Effect of 5-ALA on the expression of oxidative stress-related genes during HS. Bovine MECs were pretreated with or without 5-ALA at concentrations of 10, 100, and 500 μM for 24 h followed by HS (42.5°C for 48 h). Cells that were consistently cultured at 3742.5°C and received no 5-ALA treatment were used as the control group. mRNA levels of *NRF2*, *HO-1*, and *NQO1* were determined by RT-qPCR and normalized to *ACTB* levels. Data are presented as mean±standard error of the mean for three independent experiments. 5-ALA, 5-aminolevulinic acid; HS, heat stress; MEC, mammary epithelial cells; *NRF2*, nuclear factor erythroid derived 2 like factor 2; *HO-1*, heme oxygenase-1; *NQO1*, NAD(P)H quinone oxidoreductase 1; RT-qPCR, real-time quantitative polymerase chain reaction; *ACTB*, β-actin. ^a–d^ Means with different letters are significantly different, p<0.05.

We examined the effect of pretreatment with 5-ALA on the expression of nuclear factor erythroid-derived 2-like factor 2 (NRF2) and downstream signaling antioxidant genes *HO-1* and NAD(P)H quinone oxidoreductase 1 (*NQO1*) under HS. Pretreatment of bovine MECs with 5-ALA induced dramatic mRNA upregulation of *NRF2*, *HO-1*, and *NQO1* (Figure 3). 5-ALA likely provides a cytoprotective effect via upregulating NRF2 along with downstream genes *HO-1* and *NQO-1* expression.

### 5-ALA reduces HS-induced ER stress

**Figure 4:**
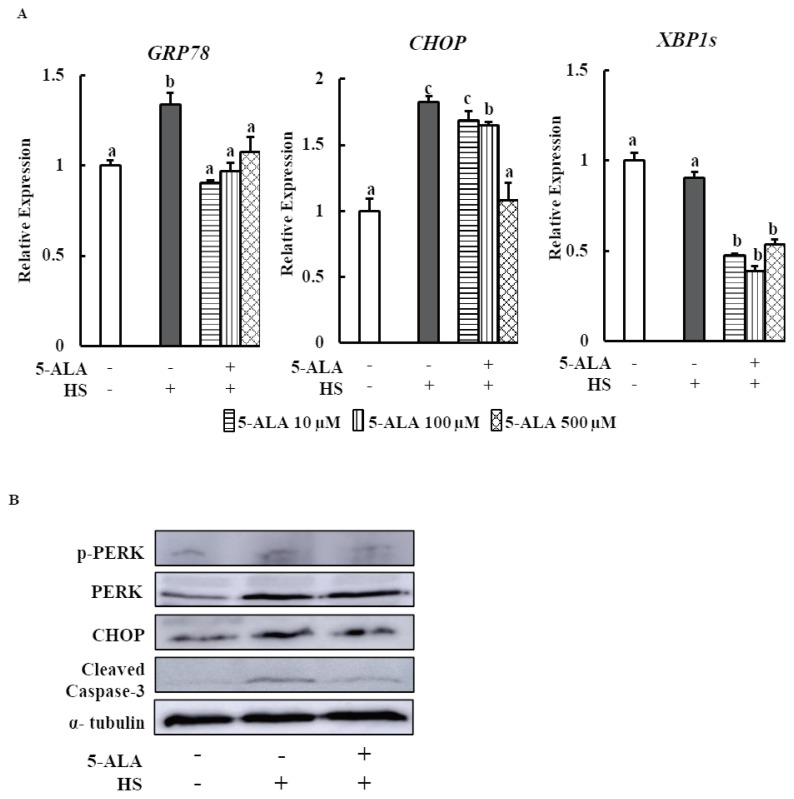
5-ALA reduces HS induced ER stress. Bovine MECs were pretreated with or without 5-ALA at concentrations of 10, 100, and 500 μM for 24 h followed by HS (42.5°C for 24 h). Cells that were consistently cultured at 37°C and received no 5-ALA treatment were used as the control group. (A) mRNA levels of *GRP78*, *CHOP*, and *XBP1s* were determined by RT-qPCR and normalized to *ACTB* levels. Data are presented as mean± standard error of the mean for three independent experiments. ^a–c^ Means with different letters are significantly different, p<0.05. (B) The phosphorylated PERK, PERK, CHOP, cleaved caspase3, and α-tubulin (internal control) protein levels were detected using Western blotting. Representative images from three independent experiments with at least four replicates in each experiment are shown. 5-ALA, 5-aminolevulinic acid; HS, heat stress; ER, endoplasmic reticulum; MEC, mammary epithelial cells; *GRP78*, glucose-regulated protein 78; *CHOP*, C/EBP homologous protein; *XBP1s*, X-box binding protein 1 splicing form; PERK, PKR-like endoplasmic reticulum kinase.

Finally, the expression of *GRP78*, a central regulator of ER stress, and *CHOP*, a key signaling component involved in ER stress-induced apoptosis, and *XBP1s*, a key transcriptional gene involved in UPR, were examined as ER stress markers in bovine MECs after pretreatment with 5-ALA. 5-ALA pretreatment significantly suppressed HS-induced *GRP78* and *CHOP* expression levels and decreased *XBP1s* expression (Figure 4A).

We performed Western blot analysis to measure phospho- PERK, CHOP, and cleaved caspase-3 protein expressions to confirm the beneficial effects of 5-ALA on bovine MECs. 5-ALA pretreatment suppressed HS-induced phospho-PERK, CHOP, and cleaved caspase-3 protein expressions (Figure 4B).

## DISCUSSION

A number of studies have been suggested that the major cause of HS-induced cellular impairment is the ER stress mediated cellular apoptosis [7,11]. But the cellular and molecular events underlying the effect of HS on bovine mammary epithelial cells remain poorly uncovered. Our study showed that HS increased the mRNA expression of *GRP78* and *CHOP* and protein expression of CHOP, p-PERK, and cleaved caspase-3 in bovine MECs (Figure 4). The major ER stress chaperon GRP78, plays a vital role in maintaining the ER homeostasis. In non-stressed cells, three UPR transducers remain as inactive form through binding with GRP78. Upon ER stress, all three sensors are released from GRP78. In this case, PERK is activated [23], subsequent transcription factor CHOP and major apoptosis executioner caspase-3 is upregulated [24]. Previous study indicated that hyperthermia increases the GRP78 and caspase-3 protein expression in human osteosarcoma and bovine granulosa cells [25,26]. It has been demonstrated that HS activates PERK-eIF2α-ATF4-CHOP pathway that enhances ER stress-mediated apoptosis in different types of cell including bovine MECs [7].

We also found that anti- and pro-apoptosis gene *BAX* and *BCL2* expressions were enhanced and suppressed respectively by HS in MAC-T cells, which is consistent with the results of Jin et al [7]. It is well established that during ER stress condition, CHOP upregulates the expression of BAX and downregulates the expression of BCL2 expression [27]. Thus, CHOP is responsible for heat treatment mediated ER stress induced cell death. Surprisingly, *XBP1s* expression did not differ between control and HS-treated bovine MECs in the present study (Figure 4A). In brief, current data postulated that HS induced UPR signaling and ER stress mediated apoptosis in bMECs. Therefore, HS has the adverse effect on mammary gland physiology of dairy cows.

Findings from this research showed that Nrf2 mRNA upregulation was higher in bMECs at all pretreatment concentrations of 5-ALA followed by HS. Nrf2 acts as a master regulator of cellular antioxidant response, which protects against oxidative stress-induced DNA damage and apoptosis [28]. Previous study demonstrated that 5-ALA is an enhancer of heme synthesis, which reduces cardiomyocyte hypertrophy via Nrf2 activation [22]. In response to oxidative stress, Nrf2 induces cellular antioxidant defense enzymes, such as HO 1 and NQO1 [29]. Further, data from this study showed that bovine MECs pretreated with 5-ALA significantly increased the mRNA expression of HO-1 and NQO1 (Figure 3). 5-ALA showed a renoprotective effect via inducing HO-1 expression in response to renal ischemia-reperfusion injury and cisplatin nephropathy in the mouse kidney [19,20]. Another research showed that enhanced expression of Nrf2 mediated NQO1, a cytosolic flavoprotein, facilitates cytoprotection against oxidative stress induced DNA damage and the apoptosis of muscle cells by suppressing intracellular ROS [30]. 5-ALA thus provides a cytoprotective effect via upregulating Nrf2 that in turn upregulates downstream antioxidant genes, *HO-1* and *NQO-1* expression, and increases the viability of heat stressed bovine MECs. Interestingly, our study also indicates that HS slightly activates Nrf2/HO-1 signaling in bMECs even without 5-ALA treatment, suggesting a self-defense mechanism in bMECs during HS. These findings are consistent with the study of Jin et al [7], who showed that Nrf2-ARE signaling is also slightly activated in heat-stressed bMECs. However, 5-ALA refrain the cell from HS induced apoptosis via increasing the Nrf2 and antioxidant genes expression.

Moreover, the present study showed that 5-ALA treatment may prevent HS-induced apoptosis of mammary epithelial cells because 5-ALA pretreatment significantly suppressed HS-induced *GRP78*, CHOP, caspase-3, and *BAX* expression and enhanced *BCL2* expression (Figures 2, 4). The key regulator of ER stress transducers, GRP78 reduction by 5-ALA may restore the basal level of p-PERK. Thereby, 5-ALA may enhance the cell viability by decreasing the CHOP expression of heat stressed cell and also simultaneously regulating the anti- and pro-apoptotic gene expression in this study. Therefore, 5-ALA can ameliorate the HS induced ER stress and raise the cell viability as well as shows the beneficial effect for udder environment of dairy cows.

In summary, our results indicate that 5-ALA provides cytoprotection via inhibition of apoptosis markers in HS-induced damage of bovine MECs. Moreover, this study demonstrates that antioxidant actions of 5-ALA are caused by the upregulation of NRF2 and its downstream antioxidant gene such as *HO-1*, *NQO1*. Overall, our results showed that the upregulation of antioxidant gene and reduction of ER stress by 5-ALA could attenuate HS-induced cell damage.

## Figures and Tables

**Table 1:** Sequences of primers used for real-time polymerase chain reaction amplification

Gene	Primers (5′ to 3′)
*XBP1s*	Forward TGCTGAGTCCGCAGCAGGTG Reverse GCTGGCAGACTCTGGGGAAG
*GRP78*	Forward GATTGAAGTCACCTTTGAGATAGATGTG Reverse GATCTTATTTTTGTTGCCTGTACCTTT
*CHOP*	Forward CTGAAAGCAGAGCCTGATCC Reverse GTCCTCATACCAGGCTTCCA
*BAX*	Forward TGGACATTGGACTTCCTTCG Reverse CCAGCCACAAAGATGGTCAC
*BCL2*	Forward GGATGACCGAGTACCTGAACC Reverse GCCCAGATAGGCACCCAG
*NRF2*	Forward CCAGCACAACACATACCA Reverse TAGCCGAAGAAACCTCATT
*HO-1*	Forward GGCAGCAAGGTGCAAGA Reverse GAAGGAAGCCAGCCAAGAG
*NQO1*	Forward CAACAGACCAGCCAATCA Reverse ACCTCCCATCCTTTCCTC
*ACTB*	Forward CATCGCGGACAGGATGCAGAAA Reverse CCTGCTTGCTGATCCACATCTGCT

*XBP1s*, X-box binding protein 1 splicing form; *GRP79*, glucose-regulated protein 79; *CHOP*, C/EBP homologous protein; *BAX*, BCL2 associated X; *BCL2*, B-cell lymphoma 2; *NRF2*, nuclear factor erythroid derived 2 like factor 2; *HO-1*, heme oxygenase-1; *NQO1*, NAD(P)H quinone oxidoreductase 1; *ACTB*, β-actin.
